# Hydralazine-Induced ANCA Vasculitis Presenting With Pericarditis: A Novel Case and Literature Review

**DOI:** 10.1155/cric/9932632

**Published:** 2025-02-27

**Authors:** Ahmed Sami Hammami, Osejie Oriaifo, Sinda Hidri, Sukhvir Singh, Husam El Sharu, Joshua Peltz, Soroush Nomigolzar, Kunjan Udani

**Affiliations:** ^1^Department of Internal Medicine, East Carolina University, Greenville, North Carolina, USA; ^2^Department of Cardiology, East Carolina University, Greenville, North Carolina, USA

## Abstract

Hydralazine, a commonly used arterial vasodilator for managing congestive heart failure and hypertension, is known to be associated with drug-induced lupus and, less frequently, antineutrophil cytoplasmic antibody (ANCA)–associated vasculitis (AAV). Drug-induced AAV typically carries a favorable long-term prognosis and is not commonly linked to cardiovascular or ocular involvement. Pericarditis cases associated with hydralazine have not been previously reported. We present a rare case involving an 85-year-old woman on long-term hydralazine therapy, initially presenting with acute lobar pneumonia. During her hospitalization, she developed pericarditis, chemosis, and conjunctivitis in her eyes, along with cutaneous lesions described as a maculopapular rash on her face, tender bullae on her digits, and a petechial rash on her back. Laboratory findings were consistent with drug-induced AAV, showing positive myeloperoxidase and proteinase 3 antibodies. An attempted diagnostic pericardiocentesis was unsuccessful. Hydralazine was discontinued, and she was successfully treated with corticosteroids and tolerated immunosuppression well. Subsequently, she recovered and was discharged from the hospital.

## 1. Introduction

Hydralazine was introduced in the 1950s as a treatment for malaria [1]. It acts by direct vasodilation of arterioles and therefore reduces peripheral vascular resistance [2]. This drug has been used to treat moderate to severe hypertension in patients with renal dysfunction, hypertensive emergencies in pregnancy, and also heart failure with reduced ejection fraction (in the setting of angiotensin-converting enzyme inhibitor or angiotensin receptor blocker intolerance) [3]. Hydralazine-induced lupus was first described in 1953. Clinical presentation ranged from lethargy, arthralgia, myalgia, and skin rash to multiorgan involvement. The occurrence of systemic vasculitis is a very rare complication that can have a rapidly progressive and fatal course when it presents with the pulmonary–renal syndrome; however, cardiac and ophthalmologic manifestations have been seldomly described [4, 5]. Here, we report a severe complication of hydralazine-induced antinuclear cytoplasmic antibody (ANCA)–associated vasculitis (AAV) presenting as pericardial effusion and conjunctivitis.

## 2. Case Report

We report the case of an 82-year-old Caucasian female with past medical history significant for breast cancer treated with lumpectomy, moderate aortic stenosis, heart failure with preserved ejection fraction, chronic kidney disease Stage IV with associated anemia of chronic disease, Type 2 diabetes mellitus (diet controlled), essential hypertension, hyperlipidemia, and a history of hemorrhagic stroke (in the right basal ganglia in February, 2021), presenting with symptoms of shortness of breath, productive cough, low-grade fever, and lethargy for 2 weeks. The patient was found to have a left lower lobe community-acquired pneumonia complicated by left-sided parapneumonic effusion, and she was treated with 3 days of azithromycin po and 5 days of ceftriaxone IV. A left-sided thoracocentesis was performed, removing around 500 cc of fluids consistent with a transudative process. On Day 5 of hospitalization, the patient developed extreme lethargy and arthralgia and complained of new painful cutaneous lesions in her hands, forehead, and back; she also reported a blurry vision with serosanguinous tears.

### 2.1. Clinical Findings

**Figure 1:**
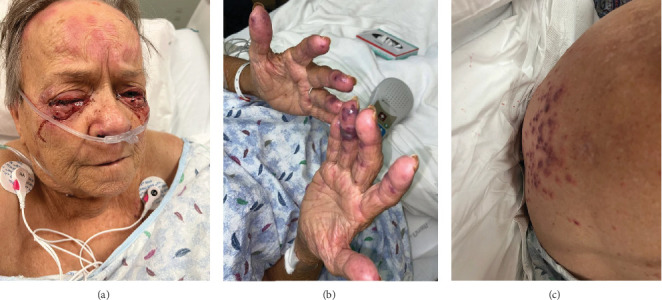
(a) Chemosis, bilateral palpebral edema, and conjunctivitis. Diffuse confluent maculopapular lesions are visible on the forehead of the patient. (b) Hemorrhagic bullae over multiple digits bilaterally. (c) Multiple confluent nonblanching palpable purpura in her back.

On physical examination, the blood pressure was 138/80, the heart rate was 105 beats per minute, the respiratory rate was 18 breaths per minute, the oxygen saturation (SpO2) was 95%, and the temperature was 36.7°C. The patient appeared lethargic; the head, eyes, ears, nose, and throat (HEENT) examination showed crusting over her nostrils and prominent tender cervical lymphadenopathies. The ocular exam revealed bilateral palpebral edema, subconjunctival hemorrhage, conjunctivitis, and chemosis. The skin exam revealed a maculopapular rash over her forehead, multiple confluent nonblanching palpable purpura in her back, and hemorrhagic bullae in her digits tender to palpation bilaterally (Figure 1). The cardiac examination revealed a systolic murmur grade 4/6 in the aortic area. No S3 or S4 sounds were heard. Pulmonary auscultation was consistent with rhonchi in the left lower lobe.

Of note, the patient does not have any previous personal or family history of autoimmune or inflammatory disorder. She denies smoking or drinking alcohol. She also denies using any drugs or herbal medicine. Her home medication included hydralazine, aspirin, atorvastatin, anastrozole, furosemide, gabapentin, and metoprolol.

### 2.2. Diagnostic Assessment

Laboratory data revealed a hemoglobin of 9.6 g/dL, a platelet count of 405 K/*μ*L, a white blood count of 3650/*μ*L, and an eosinophil count of 0.16 K/*μ*L. Complete metabolic panel showed a potassium of 4.6 mEq/L, creatinine was 2.69 mg/dL (the reference range is 0.57–1.11 mg/dL), and the estimated glomerular filtration rate was 16.9 mL/min per 1.73 m^2^ (a review of her renal function 3 months prior to her admission revealed a creatinine of 2.07 mg/dL, with a glomerular filtration rate of 19 mL/min/1.73 m^2^). The erythrocyte sedimentation rate was 68 mm/h, and C-reactive protein was 219.1. Creatinine kinase was 94 units/L, aldolase was high at 8.8 U/L (reference range < 7.7 U/L), brain natriuretic peptide (BNP) was high at 482 pg/mL, troponin was < 0.03 ng/mL, and thyroid-stimulating hormone (TSH) was 0.51 mIU/L (reference range 0.4–4.1 mIU/L). Complement levels were normal, with C3 at 90 mg/dL and C4 at 18 mg/dL. The antinuclear antibody (ANA) test using the HEp-2 substrate was positive, with a titer of 1:1280 and a homogeneous pattern. The patient also had a positive p-ANCA and negative c-ANCA. The myeloperoxidase (MPO) antibody was elevated at 2.6 U (referencerange < 0.4), and the proteinase 3 (PR3) antibody was 4.4 U (referencerange < 0.4). The rheumatoid factor (RF) was elevated at 26 IU/mL (reference range < 15 IU/mL). The anti-cyclic citrullinated peptide was < 15.6 U, the antihistone antibody (AHA) was < 1 U, and the cryoglobulin test was negative. The anti-double-stranded DNA (dsDNA) antibody was 26.2 IU/mL (referencerange < 30 IU/mL). The respiratory PCR panel was negative for influenza A and B and respiratory syncytial virus. Hepatitis C and B serology antibody was negative. Although clinical suspicion of TB was low but given the initial respiratory symptoms of the patient, it was ruled out using an acid-fast bacilli smear of the sputum.

Thoracocentesis fluid analysis was as follows: LDH was 115 units/L and total protein was 3 g/dL (serum LDH is 733 units/L and total serum protein is 6.7 g/dL). None of Light's criteria were met for exudative pleural effusion.

The urinalysis showed 2+ RBCs and 1–4+ WBCs. The urine creatinine level was 60.48 mg/dL, and the total urine protein was 0.04 g per 24 h (the reference range is 0.04–0.15 g per 24 h).

The electrocardiogram (EKG) showed sinus tachycardia at 103 beats per minute, a normal axis, and nonspecific ST-T wave abnormalities. There were no evident changes compared to an EKG done 2 years before.

**Figure 2:**
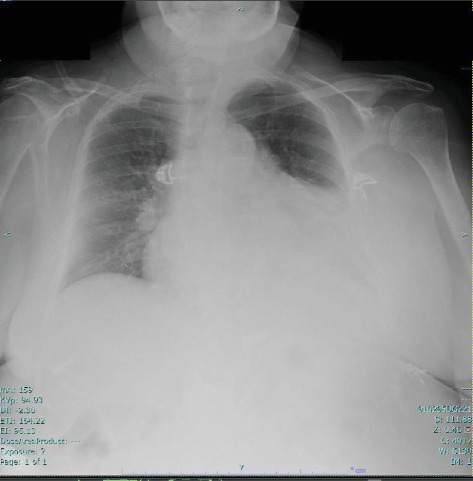
Chest X-ray shows a left lower lobe pneumonia associated with a parapneumonic effusion.

The chest X-ray showed a consolidation in the left lower lobe associated with a parapneumonic effusion (Figure 2). A CT scan of the chest without IV contrast, performed on admission, was consistent with patchy opacity in the left lower lobe with air bronchograms and an adjacent pleural effusion, suggestive of lobar pneumonia. It also showed a moderate to large pericardial effusion.

**Figure 3:**
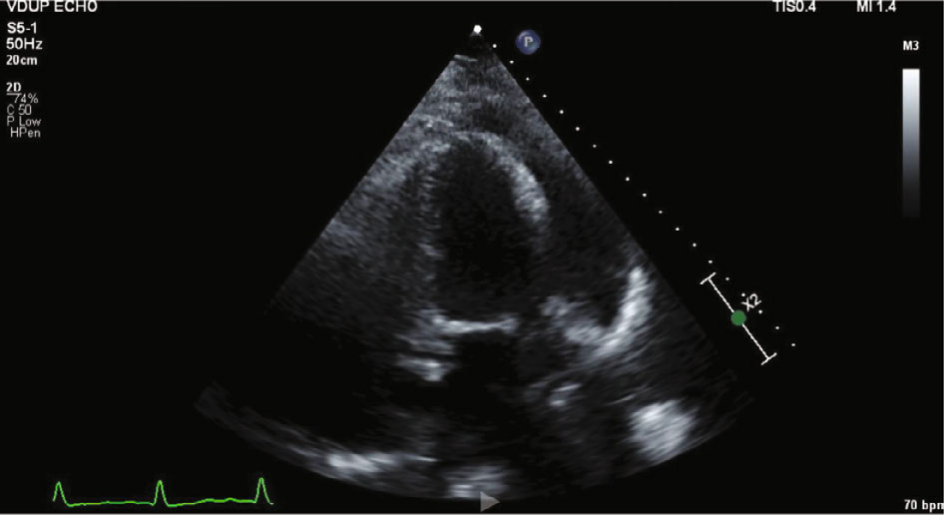
Apical Four-Chamber view on transthoracic echocardiography showing a 1–2-mm pericardial effusion.

Transthoracic echocardiogram showed a preserved ejection fraction of 55%–60%, and left and right ventricles were normal in size and function; there was moderate valvular aortic stenosis with a peak aortic valve velocity of 343 cm/s and a moderate-sized pericardial effusion measuring 1–2 cm, without echocardiographic findings consistent with tamponade (Figure 3).

The differential diagnosis is as follows:

1. Hydralazine-induced lupus
2. Hydralazine-induced vasculitis
3. Wegner granulomatosis with polyangiitis
4. Microscopic polyangiitis
5. Eosinophilic granulomatosis with polyangiitis
6. Mixed cryoglobulinemia

### 2.3. Diagnosis

Prior to the admission, the patient used to take hydralazine 50 mg three times a day for more than 2 years. Based on her risk factors including a long course and an elevated cumulative dose of hydralazine, an agent, notorious to cause drug-induced AAV, the patient was suspected to have a hydralazine-induced AAV after excluding other causes including infections, malignancies, and other types of vasculitis. No kidney biopsy was attempted as her kidney function decline quickly recovered and was thought to be most likely prerenal as it responded well to fluid. Skin biopsy was not obtained as the patient declined the procedure.

### 2.4. Treatment

Based on the diagnosis, hydralazine was therefore discontinued, and the patient was started on pulse steroids 60 mg daily for 7 days with a taper at discharge. A diagnostic pericardiocentesis was attempted but was unsuccessful. The patient's condition improved over the course of the next 7 days after discontinuation of the suspected culprit agent, which was also in favor of our diagnosis.

### 2.5. Outcome and Follow-Up

At the follow-up appointment 2 months later, repeat echocardiography showed the resolution of the pericardial effusion, and she was continued on low-dose prednisone 5 mg per day.

Over the course of the next year, the patient developed a new left heel nontraumatic ulcer for which she underwent surgical debridement and resection. This was thought to be related to her vasculitis; therefore, she was started on avacopan. Also, her kidney function continued to decline and she required peritoneal dialysis, and at the time of writing this case report, the patient has been referred to obtain vascular access, for hemodialysis.

## 3. Discussion

AAV is a rare clinical entity. It comprises multiple vasculitis including Wegener's granulomatosis, microscopic polyangiitis, and Churg–Strauss syndrome. The overall annual incidence is approximately 10–20 cases per million, and the peak age of onset is 65–74 years old according to Ntatsaki, Watts, and Scott [6]. Medication-induced vasculitis was reported in scientific papers as early as the 1940s. This was later endorsed by the discovery of ANCA and their target antigens such as PR3 and MPO in the 1980s, with numerous case series describing patients who were ANCA positive after exposure to medications, such as allopurinol, hydralazine, propylthiouracil (PTU), procainamide, methotrexate, and minocycline [7]. This usually leads to the generation of autoantibodies and a clinical autoimmune picture. The pathogenesis associated with hydralazine-induced AAV is hypothesized to be related to (1) hydralazine binding to MPO leading to neutrophil apoptosis; (2) hydralazine-triggered disruption of epigenetic silencing of MPO, resulting in heightened expression of autoantigens in neutrophils; (3) variation in hydralazine metabolism leading to a loss of tolerance in individuals with slow acetylation compared to fast acetylators, causing distinct immune responses [5]. Yokogawa and Hawn conducted in 2009 an extensive literature review where they reported 68 cases of hydralazine-induced vasculitis. The mean age was 64 years old, with a predominance of female (62%), with 81% and 19% having kidney and pleuropulmonary involvement, respectively. Arthralgia, upper airway involvement, and cutaneous vasculitis were reported in 20%–25% on average. However, none of the patients had a cardiovascular manifestation such as pericarditis and only two patients had ocular manifestation associated with a hydralazine-induced lupus [8]. The series also had serological evidence of an autoimmune process, with 96% of patients testing positive for ANA (91% exhibiting a homogeneous pattern). 26% were positive for anti-dsDNA antibodies, and hypocomplementemia with low C3 and C4 was identified in 44% of cases. Hawn et al. described in 2022 the first case where an ophthalmologic manifestation alone led to a diagnosis: The patient was an 88-year-old female who was initially admitted for lower gastrointestinal bleeding, and 9 days after admission, she developed conjunctival chemosis and injection which rapidly progressed into a grouped eruption and blisters surrounding the periorbital skin, face, and neck, which is a similar presentation to our case [9]. In our review of the current literature, no cases of hydralazine-induced vasculitis with cardiovascular involvement were reported. Pleuritis and pericarditis are well recognized in eosinophilic granulomatosis with polyangiitis (EGPA) but considered rare manifestations of other forms of AAV and have been described only sporadically in drug-induced AAV [10]. In fact, according to Thompson et al., there was a higher frequency of pericarditis in EGPA compared with that in the other AAVs (*p* < 0.01); this owes to the underdiagnosis of pericarditis, especially as inaugural manifestation of the disease [10].

Our case highlights the importance of recognizing pericardial effusion with or without major hemodynamic compromise as a possible initial manifestation of drug-induced AAV. Cardiac manifestation has been reported in EGPA with a prevalence at 66%, usually presenting as progressive perimyocarditis, myocardial infarction, or heart failure, and therefore, an extensive workup with EKG, echocardiography, cardiac imaging, and cardiac enzymes is recommended for screening and diagnosis of these manifestations and we believe the same approach is warranted for drug-induced AAV [11, 12]. Colakovski and Lorber described in 2000 the first case of PTU-induced AAV associated with pericarditis: A 25-year-old woman with Graves' disease treated with PTU for 10 months presented with a febrile illness and right upper quadrant abdominal pain, nausea, and vomiting and was found to have a small pericardial effusion on echocardiogram. Immunologic studies revealed positive p-ANCA and elevated MPO levels, the patient underwent pericardial window, and biopsy and pathology revealed granulation tissue in association with acute and chronic inflammation without granuloma. PTU therapy was withdrawn, and an anti-inflammatory regimen was started with prednisone 60 mg daily which yielded rapid resolution of her symptoms [13]. The same treatment approach was adopted in our case and led to a positive outcome.

There is a significant potential for overlap between hydralazine-induced vasculitis and hydralazine-induced lupus, including the potential involvement of pericarditis. Therefore, it is essential to exercise caution and refrain from conflating these two conditions. Yokogawa and Hawn distinguished these two different entities by identifying vasculitic symptoms, including rapidly progressing glomerulonephritis, pulmonary damage, necrotizing cutaneous vasculitis, or retinal vasculitis. Also, lab findings can highlight the diagnosis; in fact, the presence of hypocomplementemia and leucopenia can be in favor of AAV [8].

The interval between the first exposure and the occurrence of symptoms is extremely variable, ranging from few days to few years, sometimes occurring after drug dosage increases or after a severe illness such a pneumonia that we believe might have triggered our patient's symptoms [14]. According to Hawn et al.'s series, the subjects in the study had a mean exposure to hydralazine of 4.7 years with a mean dose of 142 mg/day (ranging from 50 to 250 mg/day) [9]. The longer duration of therapy and higher daily doses are considered risk factors that can lead to the disease [15, 16].

To date, there is no standard treatment strategy for drug-induced AAV. Treatment options can vary depending on the severity of the disease as outlined in Weng and Liu for patients with mild symptoms including arthralgia, fever, weight loss, and without organ involvement, stopping the offending drug at once after diagnosis may be sufficient to induce disease remission; however, in the case of active organ involvement, such as cardiac, renal, or pulmonary involvement, a more aggressive approach is sometimes warranted consisting mainly of immunosuppressive therapy such as steroids, cyclophosphamide, rituximab, and rarely plasmapheresis as shown in the MEPEX trial in severe pulmonary–renal syndrome [17]. Corticosteroids induce the suppression of T and B cell proliferation, maturation, and activation, crucial processes for the generation of anti-MPO [18, 19]. In the case of our patient, who developed cutaneous, cardiac, and ocular symptoms after 2 years of hydralazine use, her condition improved with the discontinuation of the offending agent and administration of steroids.

Compared with primary AAV, drug-induced AAV is easier to treat, but severe cases may require more aggressive immunosuppression. Generally, a shorter course is required, and in some instances, no maintenance dose is warranted. The long-term prognosis is typically favorable if the patient can withstand and tolerate the immunosuppressive treatment. Novel therapies such as avocapon, a C5a receptor inhibitor, have been shown in the ADVOCATE trial to be safe, well-tolerated, and noninferior to prednisone in the treatment of AAV by Week 26 and superior by Week 52 in achieving clinical remission. This may revolutionize AAV treatment as it may have the potential to replace our reliance on oral steroids for induction therapy.

## 4. Conclusion

This is the first reported case of hydralazine-induced vasculitis presenting with pericarditis and ocular manifestations. The patient, initially admitted for lobar pneumonia, developed leukocytoclastic vasculitis cutaneous lesions, pericarditis, and bilateral chemosis and conjunctivitis. Her symptoms and other manifestations of hydralazine-induced vasculitis improved after discontinuation of the offending agent and the start of steroids.

Although pericarditis may occur in association with other inflammatory illnesses, it remains an important feature of drug-induced AAV; therefore, a careful review of the patient's symptoms, imaging, and serological results is strongly recommended, as hydralazine-induced autoimmunity can be very severe and potentially life-threatening.

## 5. Limitations

Though the most common viral infections causing pericarditis are reported to be coxsackievirus (Types A and B) and echovirus, most of these data come from children diagnosed by serologic testing in the 1960s. More recent data suggest that adult patients are more commonly infected with cytomegalovirus and herpes viruses as well as HIV [20]. For the above case report, a full respiratory viral panel was performed, but it did not include coxsackievirus or echovirus serology.

While skin biopsy would have been beneficial to highlight the diagnosis even more, the patient has declined to consent for this procedure, and we honored her wishes. Although a drug-induced lymphocyte stimulation test and gallium-67 scintigraphy can be helpful as supplementary tools in diagnosing drug allergies and in elucidating the extent of the disease, respectively, this data was not used in the case of our patient given the inconsistent reliability of these tests [21, 22].

## Data Availability

Data can be made available upon request to the corresponding author.

## Nomenclature

AAV: antineutrophil cytoplasmic antibody–associated vasculitis
AHA: antihistone antibody
ANA: antinuclear antibody
ANCA: antineutrophil cytoplasmic antibody
MPO: myeloperoxidase
RF: rheumatoid factor
dsDNA: double-stranded DNA
EGPA: eosinophilic granulomatosis with polyangiitis
EKG: electrocardiogram
